# Neonatal Fungemia by Non-Candida Rare Opportunistic Yeasts: A Systematic Review of Literature

**DOI:** 10.3390/ijms25179266

**Published:** 2024-08-27

**Authors:** Alexandra Mpakosi, Vasileios Cholevas, Joseph Meletiadis, Martha Theodoraki, Rozeta Sokou

**Affiliations:** 1 Department of Microbiology, General Hospital of Nikaia “Agios Panteleimon”, 18454 Piraeus, Greece; 2 School of Medicine, University of Bologna, 40125 Bologna, Italy; billcholevas34@gmail.com; 3 Clinical Microbiology Laboratory, Attikon University Hospital, Medical School, National and Kapodistrian University of Athens, 12462 Athens, Greece; jmeletiadis@med.uoa.gr; 4 Neonatal Intensive Care Unit, General Hospital of Nikaia “Agios Panteleimon”, 18454 Piraeus, Greece; anastasiosmmr@yahoo.gr; 5 Neonatal Department, Aretaieio Hospital, National and Kapodistrian University of Athens, 11528 Athens, Greece

**Keywords:** neonatal fungemia, rare opportunistic yeasts, non-*Candida* infection, premature neonates, low birth weight, fluconazole prophylaxis

## Abstract

Fungal colonization poses a significant risk for neonates, leading to invasive infections such as fungemia. While *Candida* species are the most commonly identified pathogens, other rare yeasts are increasingly reported, complicating diagnosis and treatment due to limited data on antifungal pharmacokinetics. These emerging yeasts, often opportunistic, underscore the critical need for early diagnosis and targeted therapy in neonates. This systematic review aims to comprehensively analyze all published cases of neonatal fungemia caused by rare opportunistic yeasts, examining geographical distribution, species involved, risk factors, treatment approaches, and outcomes. Searching two databases (PubMed and SCOPUS), 89 relevant studies with a total of 342 cases were identified in the 42-year period; 62% of the cases occurred in Asia. *Pichia anomala* (31%), *Kodamaea ohmeri* (16%) and *Malassezia furfur* (15%) dominated. Low birth weight, the use of central catheters, prematurity, and the use of antibiotics were the main risk factors (98%, 76%, 66%, and 65%, respectively). 22% of the cases had a fatal outcome (80% in Asia). The highest mortality rates were reported in *Trichosporon beigelii* and *Trichosporon asahii* cases, followed by *Dirkmeia churashimamensis* cases (80%, 71%, and 42% respectively). Low birth weight, the use of central catheters, the use of antibiotics, and prematurity were the main risk factors in fatal cases (84%, 74%, 70%, and 67%, respectively). 38% of the neonates received fluconazole for treatment but 46% of them, died. Moreover, the rare yeasts of this review showed high MICs to fluconazole and this should be taken into account when planning prophylactic or therapeutic strategies with this drug. In conclusion, neonatal fungemia by rare yeasts is a life-threatening and difficult-to-treat infection, often underestimated and misdiagnosed.

## 1. Introduction

In recent years, there have been increasing cases of invasive fungal infections caused by rare yeasts, which comprise non-*Candida* and non-*Cryptococcus* species. These yeasts, although ubiquitous in the environment, have been defined as rare because they are almost never associated with human infections. However, these emerging species are increasingly harming human health, alarming the global scientific community. In 2022, recognizing the seriousness of this global health issue, the World Health Organization (WHO) introduced its first list of fungal “priority pathogens”: https://iris.who.int/bitstream/handle/10665/363682/9789240060241-eng.pdf, access on 31 July 2024. This important initiative highlights the urgent need for policy reforms and enhanced research efforts, especially in understanding the spread of fungal diseases, emerging patterns of antifungal resistance, and identifying the populations most at risk for these infections. A significant concern in the management of these infections is the increasing resistance to existing antifungal therapies. This trend complicates treatment and highlights the need for new and more effective antifungal agents. Additionally, there is growing recognition of the role of fungal infections in worsening outcomes, particularly in critically ill and immunocompromised patients, including neonates.

Neonates, especially premature infants, are vulnerable organisms, easily colonized by fungi due to their immature immune system, poor skin, insufficient mucosal barriers, and multiple risk factors favoring fungal prevalence against normal flora.

Invasive fungal infections (IFIs) are a significant contributor to morbidity and mortality among neonates in neonatal intensive care units (NICUs), particularly affecting preterm infants and those with very low birth weight (VLBW, <1500 g birth weight [BW]) [1]. These neonates are especially vulnerable due to their compromised immune systems, exposure to broad-spectrum antibiotics, immature epithelial barriers, and frequent need for invasive procedures, which collectively heighten their risk for opportunistic fungal infections. The bulk of IFIs are attributed to *Candida* species [2]. Notably, invasive candidiasis (IC) ranks as the third leading cause of late-onset sepsis in VLBW neonates and is a major factor in morbidity and mortality within the NICU [3,4,5,6]. The prevalence of IC in NICUs varies widely, ranging from 0.5% to 20%, and is significantly influenced by the specific center and patient demographics. It is inversely related to gestational age and birth weight, with the highest rates (5–20%) observed among neonates with extremely low birth weight (ELBW) [2,3]. Recently, a global change in the epidemiology of candidemia has been observed, marked by the emergence of resistant non-albicans *Candida* species. Among these, *C. auris* has been spreading globally, causing outbreaks in hospitals among both pediatric and adult patients, particularly in ICUs, and has been highlighted in the CDC’s report on urgent threats https://www.cdc.gov/antimicrobial-resistance/?CDC_AAref_Val=https://www.cdc.gov/DrugResistance/Biggest-Threats, access on 30 July 2024.

Fungal colonization is an important risk factor facilitating invasion, dissemination and eventually causing fungemia. Although *Candida* is the most commonly identified species of fungemia in neonates, other yeasts are also sporadically reported worldwide raising concerns due to difficulties in identification, limited pharmacokinetic data of the available antifungals, and lack of sufficient clinical and microbiological information. These rare yeasts are emerging, opportunistic pathogens that may lead to underdiagnosis of fungemia, delayed initiation of antifungal therapy, and often have fatal outcomes [4]. Therefore, an early and specific diagnosis and targeted treatment are critical, especially for neonates. To the best of our knowledge, there are no previous reviews of neonatal fungemia cases by rare opportunistic yeasts. The present study aims to provide a systematic review of all neonatal fungemia cases by rare opportunistic yeasts published in the literature.

## 2. Methods

A research methodological protocol was developed based on the PRISMA guidelines (presented as a Supplementary Material) [7] to collect and evaluate studies related to the subject under investigation. This protocol is registered in the PROSPERO database (CRD 42024561133).

### 2.1. Formulation of the Research Question

In order to clearly formulate the research question and facilitate a literature review, the PICO (Population, Intervention, Comparison, Outcome) framework was used:

- Population: Neonates
- Intervention: Rare opportunistic yeasts
- Comparison: Not required for this review
- Outcome: Cases of neonatal fungemia

### 2.2. Development of the Review Protocol

Research question: To conduct a review of all cases of neonatal fungemia from non-*Candida* rare opportunistic yeasts that have been published in the international literature.

Inclusion Criteria:

- Case reports and case series studies reporting on cases of neonatal fungemia from non-*Candida* rare opportunistic yeasts.
- Study designs focused on non-*Candida* rare opportunistic yeast infections, mainly defined as positive cultures from blood in neonates.
- Exclusion Criteria:
- Studies referring to other forms of fungal infections.
- Cases not involving neonates.
- Duplicate publications of the same cases.
- Review articles, systematic reviews, and meta-analyses. Conference proceedings will be excluded.
- Neonatal fungemia by *Candida* species

Main Outcomes:

1. Clinical presentation of non-*Candida* rare yeasts infections in neonates
2. Risk factors and characteristics of the affected population
3. Outcomes of the infections, including mortality

### 2.3. Search Strategy-Data Sources

The systematic literature review was conducted from June 2024 to July 2024. A systematic review of the existing literature was performed by searching the electronic databases PubMed and Scopus, with a final search date of 1 July 2024. The literature review was conducted using the following keywords: “neonatal fungemia”, “rare yeasts”, “non-*Candida* species”, “fungal infections”, “fungemia”, “fungaemia”, “neonates”, “preterm infants”, “preterm neonate*”, “infant*”, “newborn*”, “case report”, “case series”, “case”, in combination with Boolean operators (AND, OR). Additionally, to minimize the risk of missing studies and fully cover the extent of available literature, a manual electronic search and review of the references of each selected study, as well as references from previous systematic reviews in the same research field, was conducted.

### 2.4. Conflict Resolution

Screening, data extraction, and quality assessment were conducted independently by two researchers (AM and RS), with conflicts resolved through discussion and consensus between the researchers or, if necessary, by a third researcher (JM).

### 2.5. Data Synthesis and Presentation

Data were recorded in tabular form according to the following criteria: type of opportunistic yeasts, day of life when the infection manifested, comorbidities, risk factors, prematurity, administration of total parenteral nutrition, placement of central catheters, mechanical ventilation, and prior administration of broad-spectrum antibiotics, number of participants, number of events, and other relevant criteria for grouping the study population (subpopulations of neonates: preterm neonates, very low birth weight neonates, cardiac surgery or other surgical cases, neonates with congenital anomalies, etc.), year of publication.

## 3. Results

**Figure 1:**
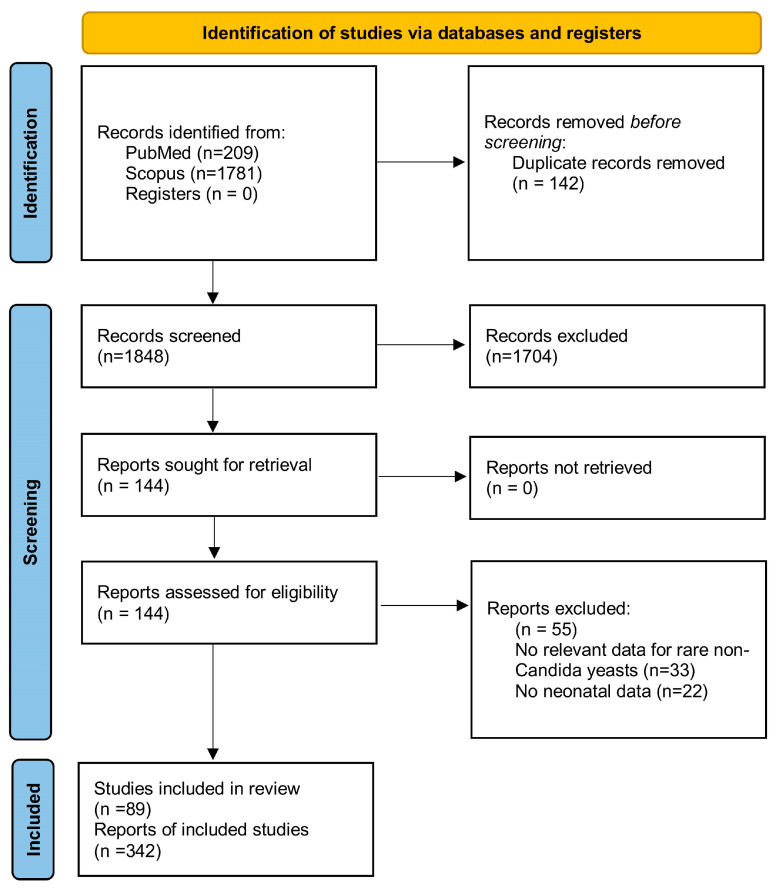
Flow diagram of the systematic review.

From the electronic database search, a total of 1990 studies were retrieved. Among these, 142 duplicate entries were identified and subsequently removed using the duplicate removal tool in the reference management software (EndNote X8). After carefully reading the titles and abstracts of the remaining studies, 1704 studies were excluded either because their subject matter did not serve the purpose of the review or because they met some of the exclusion criteria already visible at the title and abstract level. The careful reading of the full text of the 144 remaining studies revealed that only 89 studies met all the inclusion criteria and were included in this review. In total, 342 cases of neonates with rare opportunistic yeast infections were recorded. The flow diagram is presented in Figure 1.

### 3.1. Species Taxonomy and Geographical Distribution

**Figure 2:**
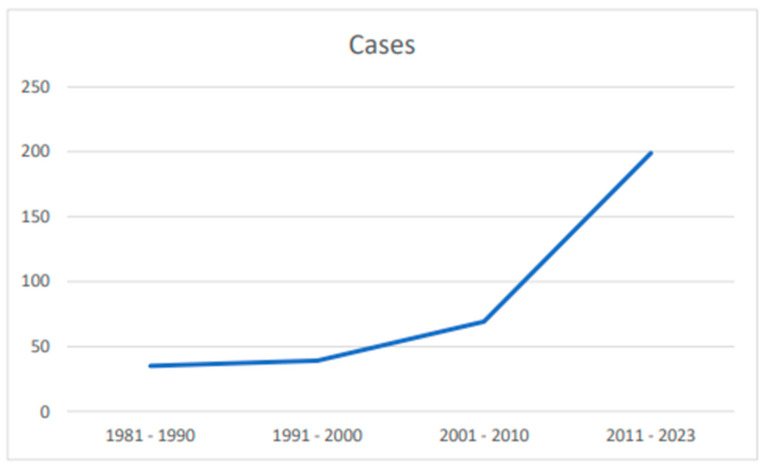
Published cases over the years.

The first report of neonatal fungemia from rare non-*Candida* yeasts was in 1981. Since then, there have been several cases and studies examining the incidence, diagnosis, and treatment of these infections. Over the past 42 years, 342 cases of neonatal fungemia caused by rare non-*Candida* yeasts have been reported. (Table 1 and Table 2, Figure 2)

**Figure 3:**
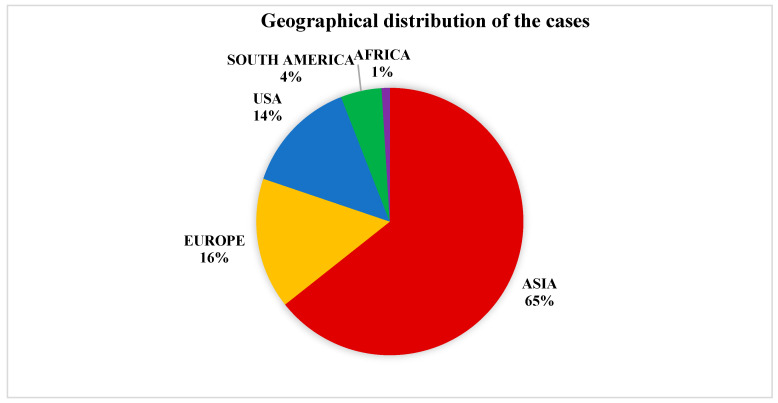
Geographical distribution of the cases.

**Figure 4:**
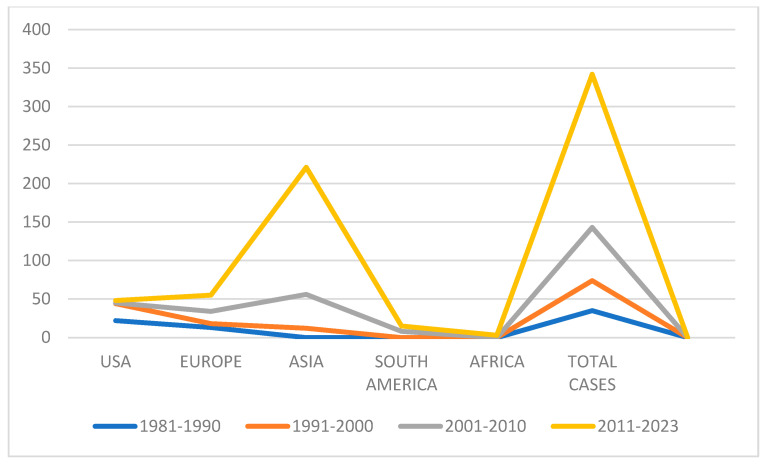
Geographical distribution of the cases over the decades.

**Figure 5:**
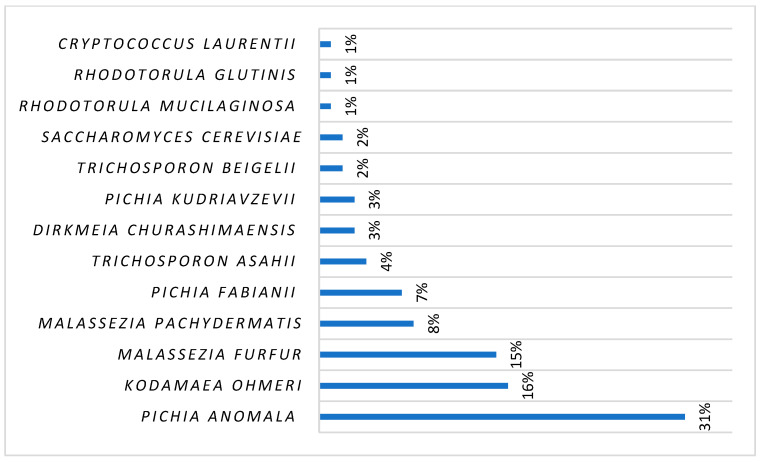
Neonatal fungemia by rare yeast species: Published data since 1981.

Regarding the geographical distribution of all reported cases, 221 (65%) were from Asia, 55 (16%) from Europe, 48 (14%) from the USA, 15 (4%) from South America, and 3 (1%) from Africa (Figure 3 and Figure 4). More specifically, *Pichia anomala* (synonyms: *Candida pelliculosa*, *Hansenula anomala*, *Wickerhamomyces anomalus*)was isolated in 108 (31%) cases (82% in Asia), *Kodamaea ohmeri* (previously known as *Pichia ohmeri*) in 54 (16%) cases (94% in Asia), *Malassezia furfur* in 51 (15%) cases (48% in USA, 48% in Europe), *Malassezia pachydermatis* in 29 (8%) cases (55% in USA), *Pichia fabianii* (synonym: *Cyberlindnera fabianii*) in 25 (7%) cases (84% in Asia), *Trichosporon asahii* in 14 (4%) cases (92% of the cases were in Asia), *Dirkmeia churashimaensis* in 12 (3%) cases (100% in Asia), *Pichia kudriavzevii* in 9 (3%) cases (100% in Asia), *Trichosporon beigelii* in 6 (2%) cases (83% in USA), *Saccharomyces cerevisiae* in 6 (2%) cases (67% in Asia), *Rhodotorula mucilaginosa* in 5 cases (1%) (80% in Europe), *Cryptococcus laurentii* (synonym: *Papiliotrema laurentii*) in 5 (1%) cases (75% in Asia), *Rhodotorula glutinis* in 4 (1%) cases (100% in Asia), *Trichosporon mucoides* in 3 cases (100% in Asia), *Moesziomyces* (*Pseudozyma) aphidis* in 2 cases (in Asia and in Europe), *Lodderomyces elongisporus* in 2 cases (in Latin America and in Asia), *Trichosporon* spp. in one case in Europe, *Malassezia sympodialis* in one case in South America, *Moesziomyces (Pseudozyma) bullatus* in one case in Africa, *Trichosporon asteroides* in one case in Europe, *Cryptococcus neoformans* in one case in Asia, *Aureobasidium melanogenum* in one case in Asia, and *Cryptococcus terreus* in one case in Europe (Figure 5).

### 3.2. Risk Factors

**Figure 6:**
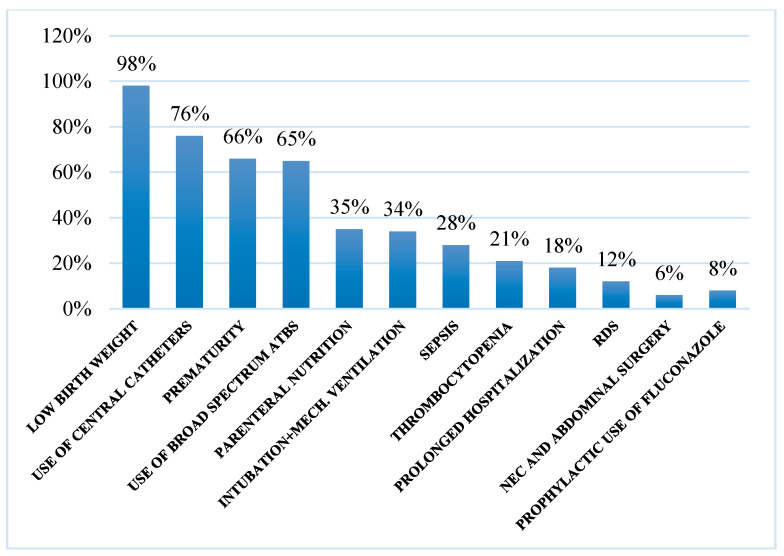
Reported risk factors. (Intubation + mech. Ventilation: Intubation + mechanical ventilation, RDS: Respiratory distress syndrome).

Low birth weight, the use of central catheters, and prematurity are the three dominant risk factors for fungemia development (Figure 6).

### 3.3. Outcome

**Figure 7:**
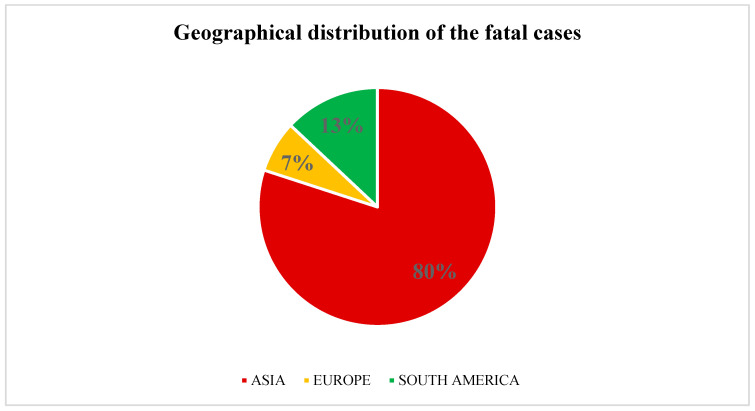
Mortality rates by continent.

**Figure 8:**
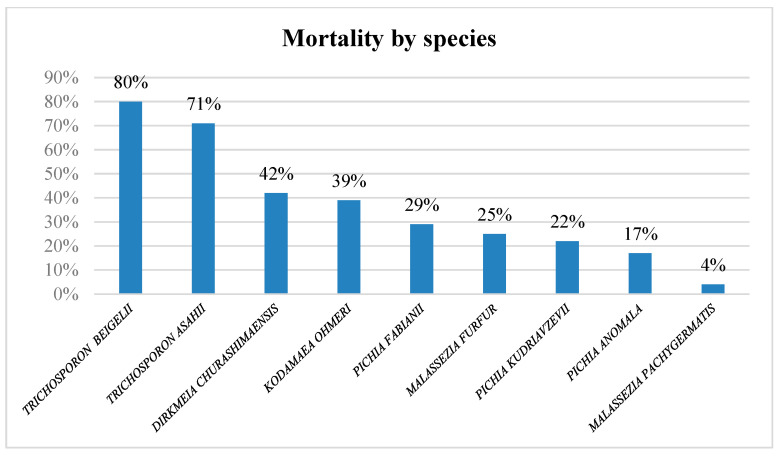
Mortality rates by species.

In 12% of the cases, the outcome was not reported. Among the remaining cases, 22% of the neonates, died. Moreover, 80% of the cases with a fatal outcome were reported from Asia (Figure 7). The highest mortality rates were noted in cases of fungemia caused by the *Trichosporon* species (Figure 8).

Neonates with Moesziomyces bullatus, Aureobasidium melanogenum, and Lodderomyces elongisporus fungemia also died.

The following risk factors were most commonly reported in total cases, compared to those associated with survival or exitus (Table 3).

### 3.4. Antifungal Susceptibility

Antifungal susceptibility testing was not reported in all cases. Nevertheless, the available data are presented in Table 4.

### 3.5. Treatment

**Figure 9:**
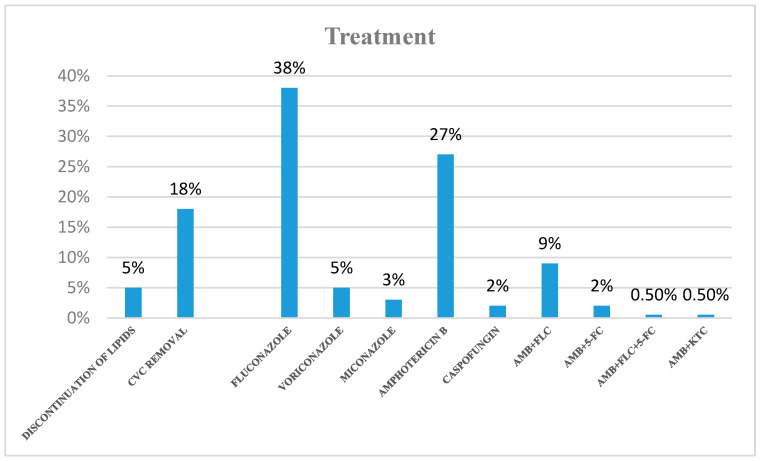
Treatment of fungemia cases (from available data). Amphotericin B (AmB), Fluconazole (FLC), Voriconazole (VOR), Miconazole (MIC), Caspofungin (CAS), 5-FC (5-flucytosine), KTC (ketoconazole).

The antifungal treatments varied notably among the cases, with Fluconazole and Amphotericin B being the most frequently administered medications. Timely removal (or replacement) of central venous catheters within 24 h of a positive blood culture is a crucial aspect of treatment. Conversely, delayed removal or replacement of central venous catheters in neonates with fungemia has been linked to higher mortality and morbidity. Our review and the available data indicated that only 18% of catheters were removed as part of the treatment intervention (Figure 9 and Table 5).

Most yeasts of this review have been recognized for decades as fungal contaminants but not as human pathogens. Systemic infections caused by them have been considered to be rare and most published studies to date are sporadic cases from all over the world. According to the data above, the majority of neonatal fungemia cases were reported in Asia, where developing countries often have low socio-economic conditions and living standards, with shortages in resources, infrastructure, and modern methods of diagnosing infections, as well as insufficient surveillance, legislation, infection control measures, and inadequately trained medical personnel who lack sufficient information about rare fungi [96]. To the best of our knowledge, from 1981 to 1990 no cases of neonatal fungemia from Asia were published and this is probably explained by the above.

Fluconazole prophylaxis is recommended worldwide in preterm infants < 1000gr from birth and until they no longer require central or peripheral catheters, as it is assumed to decrease mortality [96]. According to the respective guidelines, a third-generation cephalosporin therapy, duration of treatment with a systemic broad-spectrum antibiotic for more than 10 days, fungal colonization, and the use of a central venous catheter, are additional factors for prophylactic administration of fluconazole in such vulnerable organisms [97].

A previous systematic review and meta–analysis showed that prophylactic administration of fluconazole in different regimens may reduce mortality. Furthermore, prophylactic administration of the same regimens of fluconazole in ELBW neonates may reduce the incidence of mortality due to invasive fungal infections [98]. In addition, amphotericin B is not commercially available in 42 developing countries and is not licensed in 22 countries, making fluconazole an effective alternative therapy [96]. Nevertheless, the overuse of fluconazole in these countries, as a cheap prophylaxis in preterm low birth weight neonates, may contribute to infection development by fluconazole-resistant species [96,99]. The current study highlighted, in agreement with previous findings, the high fluconazole-resistant rates of rare yeast species such as *Pichia* spp., *Kodamaea ohmeri*, *Trichosporon* spp., *Rhodotorula* spp., *Saccharomyces cerevisiae*, etc. [100,101]. Therefore, the isolates of the present review showed elevated fluconazole MICs. This may explain why fluconazole was not the appropriate antifungal agent against those uncommon yeast species, despite the fact that it had been preferred as a treatment in 38% of all the fungemia cases. Unfortunately, 46% of the neonates who had received fluconazole therapy died. Therefore, the prophylactic use of fluconazole in neonatal intensive care units (NICUs) should be done with care to avoid resistance development and the subsequent selection of resistant species or species with reduced azole susceptibility [101]. Alternatively, nystatin is inexpensive, well-tolerated, safe, and so far with good results when prophylactically administered to preterm infants with risk factors [102,103].

## 4. Discussion

In our review the highest mortality was revealed in *Trichosporon* fungemia cases: 80% by *T. beigelii* and 71% by *T. asahii*. Previous studies have demonstrated that *Trichosporon* species were more competent than other basidiomycetes such as *Rhodotorula* spp. and *Cryptococcus* spp., to produce dense biofilms, resistant to triazoles or amphotericin B [104,105,106]. This seems to agree with our results in which all the neonatal fungemia cases by *Rhodotorula* spp. and *Cryptococcus* spp., showed zero mortality rates, despite their ability to produce biofilms on the medical devices. Previous research revealed the isolation of *Trichosporon* spp. from the hospital environment and the skin of premature neonates. Moreover, *T. beigelii* (*T. cutaneum*) was more often reported in these older studies as the causative organism of invasive infections. Nevertheless, *T. asahii* is now recognized as the dominant cause of invasive trichosporonosis threatening patients in immunosuppression and risk factors, such as neutropenia, prematurity, AIDS, extensive burns, use of catheters, broad-spectrum antibiotics or corticosteroids, mechanical ventilation, heart valve surgery, etc. Trichosporonosis is a difficult-to-treat infection due to the frequent resistance of yeast, as well as to amphotericin B, fluconazole, and combinations of the two. Nevertheless, voriconazole, posaconazole, and ravuconazole seem to act more effectively against *Trichosporon* species [105].

According to our data, *P. fabianii* fungemia cases demonstrated 29% mortality. *P. fabianii* (*Candida fabianii*, *Cyberlindnera fabianii*) is an opportunistic, emerging yeast species and may cause invasive bloodstream infections often with fatal outcomes, mainly due to its characteristic ability to produce biofilms causing resistance to antifungals [107]. Previous studies reported that *P. fabianii* may develop resistance to fluconazole, voriconazole, caspofungin, and amphotericin B [108,109].

*P. anomala* (*Wickerhamomyces anomalus*) is a plant pathogen and opportunistic cause of fungemia in both immunocompetent and immunocompromised patients. In our study, 17% of the cases had a fatal outcome. *Pichia anomala* has previously been shown to exhibit variable antifungal susceptibility with fluconazole resistance predominance [110,111]. This seems to agree with our findings and must be taken into account in NICUs for effective antifungal prophylactic and therapeutic strategies.

Fungemia by *P. kudriavzevii* (*Candida krusei*) is another life-threatening and difficult-to-treat infection due to its intrinsic resistance to fluconazole and the ability for rapid resistance development to other antifungal drugs. Vulnerable patients suffering from gastrointestinal diseases, who had previously received antibiotics, especially carbapenems, and prophylactic fluconazole, are at greater risk of developing invasive infection. In addition, whenever outbreaks occurred, sink traps and surfaces, intravenous dextrose multi-electrolyte infusion bottles, suction devices, pediatric emergency wards, and hands of health care personnel were reported as the most common sources of transmission [110]. Moreover, this species is able to produce a robust biofilm, formed by multiple layers of pseudohyphae in the polymer matrix [111]

All 12 of the neonatal fungemia cases by *Dirkmeia* occurred in the same NICU of a multispecialty hospital in Delhi with a 42% mortality rate (5/12). Surveillance cultures were obtained, but the source of infection was not identified. Infection control measures appeared to be effective as no further cases of *Dirkmeia* fungemia were reported [82]. All the twelve isolates of *Dirkmeia* had high minimum inhibitory concentrations for echinocandins and low minimum inhibitory concentrations for azoles, amphotericin B, and flucytosine.

39% of the neonates who developed fungemia by *K. ohmeri*, died. Chakrabarti et al. showed that prolonged hospitalization, piperacillin-tazobactam administration, endotracheal intubation, and mechanical ventilation, were major risk factors for the fungemia development by this rare yeast. They also demonstrated that the patients with *K. ohmeri* fungemia had significantly higher (50%) mortality than other groups of patients (some of them with *C. tropicalis* fungemia and others without fungemia), mainly due to the virulence mechanisms of the yeast species [61]. The identification of *K. ohmeri* in the laboratory is a challenge. On *Candida* chromogenic agar the color of *K. ohmeri* colonies changes from pink to blue within 48 h. Usually, species identification is performed by automated identification systems such as API 20C, Vitek 2 ID YST, and Microscan with molecular methods. For molecular identification PCR amplification is used followed by sequencing of 18S rRNA, the D1/D2 domains of 26SrRNA, the internal transcriber spacer 1/2 (ITS) of the ribosomal DNA,28SrRNA, and/or 5.8SrRNA. The pulsed field gel electrophoresis for karyotyping has been also used. Restriction endonuclease analysis of NotI-digested DNA (REAG-N) is used for genotyping of the clinical isolates of *K. ohmeri*. Fluorescent amplified fragment length polymorphism was used by Chakrabarti et al. for molecular typing. Moreover, in their study, *K. ohmeri* strains presented high fluconazole MICs [61].

Among all the *Malassezia* spp. fungemia cases, *M. furfur* dominated, from which 25% had a fatal outcome, followed by *M. pachydermatis*, with 4% mortality, and one case of *M. sympodialis* (the newborn survived). It was previously demonstrated that *M. furfur* presents high MICs to azoles. Therefore, the administration of prophylactic fluconazole in patients with risk factors such as prematurity, may lead to resistant strains and *M. furfur* colonization [112]. In catheter-related neonatal colonization by *M. furfur*, the removal of the catheter and discontinuation of intravenous lipid administration are usually sufficient and effective strategies. Furthermore, it is demonstrated that in life-threatening *M. furfur* fungemia, the preferred antifungal drug is amphotericin B [79]. In this review, *M. furfur* isolates showed elevated MICs for echinocandins.

This study also revealed the first case of neonatal fungemia due to *Aureobasidium melanogenum. Aureobasidium* species can survive in extreme ecological conditions, usually found in wet, oligotrophic environments, while their natural habitats are often subjected to osmotic stress and in many cases to solar radiation. *Aureobasidium* species can also survive on wet surfaces such as in bathrooms and saunas, in hospital environments, and can colonize organs and human skin [113]. Wang M et al. compared the two species *A. pullulans* and *A. melanogenum* and found that *Aureobasidium melanogenum* showed significantly better survival at 37 °C than *A. pullulans*, possibly due to its response to elevated temperatures with elevated melanin production which *A. pullulans* did not. This mechanism probably enhanced the virulence and pathogenicity of *A. melanogenum* [113]. This is why its accurate and timely identification is key to proper treatment as *A. melanogenum* also exhibits significant antifungal resistance. Unfortunately, *A. melanogenum* can be easily confused with *Candida* species on Gram-stained smears, while it cannot be easily identified by conventional diagnostic methods, such as VITEK 2 and MALDI-TOF MS, leaving molecular techniques as the only diagnostic selection [94].

It has recently been suggested that climate change and particularly global warming have affected the ecosystem microbiome with dramatic effects on human health. High temperatures and heat waves can lead to microbes adapting, making them thermotolerant and allowing them to survive at the human body temperature. This is particularly important for environmental fungi, most of which grow best below 37 °C. According to this view, more fungal species such as *Aureobasidium melanogenum* with resistance to environmental stress and increased pathogenicity will appear more and more frequently due to the global climate crisis threatening vulnerable organisms [114]. The same hypothesis has been made for *Candida auris*, which probably acquired the property of heat resistance due to global warming, which transformed it from an environmental species to an opportunistic human pathogen [115].

In this study, two fatal neonatal fungemia cases from another opportunistic yeast pathogen, *Lodderomyces elongisporus*, were also included. Its close relationship with *C. parapsilosis* confuses correct diagnosis, delaying treatment [92,93]. Clinical isolates of *L. elongisporus* have previously been misidentified as *C. parapsilosis* by conventional methods such as API 20C, ID 32C, and Vitek 2, while MALDI-TOF MS and molecular methods were required for correct identification [116]. However, it has been suggested that CHROMagar can be used as a preliminary identification method in laboratories where molecular methods are not available as on this agar isolates of *L. elongisporus* form turquoise blue colonies instead of the white to light pink colonies of *C. parapsilosis* [117]. This is particularly important for the timely selection of the correct antifungal therapy, as echinocandins appear to be quite effective for treating *L. elongisporus* infections but not for *C. parapsilosis* infections mainly due to the different amino acid sequence of beta-1,3 glucan synthase, which is the target of this class of antifungals [116].

This study, also showed that low birth weight, the use of central catheters, prematurity, and the use of broad-spectrum antibiotics constituted the strongest risk factors for neonatal fungemia cases as well as for the cases with fatal outcomes. It is, after all, recognized that birth weight is the major predictor of nosocomial infection development and our results are in accordance with this [118].

The birth process plays a major role in neonatal colonization by fungi. During the natural delivery, the neonatal skin is directly colonized from the maternal vaginal mycobiome. It has been previously shown that this mode of delivery is related to high colonization of *Malassezia* [119]. On the other hand, premature neonates, are mainly born via cesarian section and are thus colonized by maternal skin and the hospital environment. It has also been shown that the babies are immediately colonized by *Malassezia* species after birth with a predominance of *Malassezia furfur*, *Malassezia sympodialis*, and *Malassezia restricta* [119]. Moreover, it has been found that the neonatal gut mycobiome is already shaped at the age of 10 days with a prevalence of *Candida albicans* and *Malassezia* spp. Furthermore, *Saccharomyces cerevisiae* has been found to be the dominant fungal species in the gut mycobiome of both mothers and their 1–2-year-old babies [120]. In addition, the gut microbiome of a premature newborn contains a higher proportion of fungi than an adult’s. Moreover, factors associated with prematurity, such as an immature gastrointestinal system and intestinal barrier, an underdeveloped immune response, administration of antibiotics, and antifungal prophylaxis, can cause hematogenous dissemination of fungi and fungemia [121,122]. The type of prophylactic antifungal drug plays an important role in the composition of the gut mycobiome: a prophylactic administration of azoles may lead to prevalence of *Candida glabrata* and other azole-resistant species, while a prophylactic echinocandin strategy may cause prevalence of *Candida parapsilosis* or other echinocandin resistant species [123,124].

Neonatal infections within 48 h of birth are therefore associated with pathogens transmitted from the mother’s vagina. Other modes of transmission of microorganisms to neonates are by contact with a person or a contaminated source, droplets, or airborne transmission. Fungi are widely distributed in the environment and thus can be transmitted very easily by human agents or inanimate objects and threaten the lives of such vulnerable patients. In addition to the immature immune system, skin, or mucosal barriers, other risk factors for the development of life-threatening neonatal infections such as fungemia, include the use of catheters, invasive procedures, steroid administration, prolonged hospitalization, mechanical respiratory support, prolonged parenteral nutrition, necrotizing enterocolitis, etc. [118,121]. The colonization of the catheter or the skin at the insertion site, contamination of intravenous fluids, low frequency of catheter tubing changes, or catheter replacement may also predispose individuals to bloodstream infections. Removal of central venous catheters is therefore critical to the outcome of fungemia [123]. Moreover, it has been previously reported that the retention of a catheter is associated with increased mortality rates in *Candida* bloodstream infections [124]. The strong association between not removing the catheter and the fatal outcome was also revealed in the present review.

According to our findings, intubation and mechanical ventilation have been found in 34% of all the fungemia cases and in 65% of the fatal cases. Mechanical ventilation is recognized as a risk factor for bloodstream infections due to colonization of humidified air, or due to traumatism from the endotracheal tube and its suctioning [125]. Prolonged hospital stay also dominated in the cases with lethal outcomes (43%) compared with total cases (18%). This is understandable because a prolonged hospital stay means prolonged presence of indwelling catheters, performance of invasive procedures, and prolonged use of broad-spectrum antibiotics that favor the colonization of fungal pathogens [126]. Total parenteral nutrition and intralipids may also predispose to fungemia due to the risk of contamination or translocation of pathogens across the immature neonatal gastrointestinal mucosa and bloodstream dissemination [127]. Moreover, intralipids are predispose to infections by *Malassezia* spp. [118].

Regarding treatment management, our systematic review revealed that antifungal therapies varied significantly among cases, with Fluconazole and Amphotericin B being the most commonly administered medications. Timely removal (or replacement) of central venous catheters within 24 h of a positive blood culture is a critical component of effective treatment. Conversely, delayed removal or replacement of central venous catheters in neonates with fungemia has been associated with increased mortality and morbidity [128]. Our review and the available data showed that only 18% of catheters were removed as part of the treatment intervention. A multicenter prospective study involving 13 neonatal intensive care units was conducted in Italy to evaluate the incidence of bacterial sepsis and invasive fungal infections, fungal colonization, risk factors for sepsis and mortality rates in neonates and infants under 3 months old undergoing major surgery. They concluded that preventive measures, including early removal of vascular catheters and the use of fluconazole prophylaxis, should be implemented to mitigate the risk of bacterial and fungal infection in infants undergoing abdominal surgery, especially those with fungal colonization [129].

As mentioned above, fungal infections from fungi that were previously not considered pathogenic have been reported more and more frequently in recent years, which was also evident in our study. Given the rapid development of multidrug resistance of these emerging fungi, these infections are difficult to manage and are often associated with high mortality rates. Several studies have hypothesized an environmental pathway for this development of fungal drug resistance. For example, the widespread use of antifungals in agriculture may have caused rapid species evolution and selection of resistant strains [130]. Such opportunistic fungi can then especially threaten immunocompromised individuals including neonates.

Interestingly, our study revealed a steady increase in reported cases from 1991 to 2000, followed by a near tripling of cases from 2001 to 2023. The increase in neonatal fungemia is partially attributed to the enhanced survival rates of very low birth weight (VLBW) and extremely low birth weight (ELBW) infants. This progress has been achieved due to the regionalization of perinatal care, a better understanding of the pathophysiology of extremely premature neonates, the administration of antenatal steroids, and the critical use of postnatal surfactant therapy. Surfactant therapy has significantly improved outcomes in preterm infants by reducing the incidence and severity of neonatal respiratory distress syndrome (RDS), a common complication in very preterm infants. The introduction of surfactants has enhanced lung function, decreased the need for mechanical ventilation, and improved overall survival rates. Advances in surfactant formulations and administration techniques have further optimized its effectiveness [130]. Additionally, the use of intravenous nutrition and ongoing technological advancements have contributed to the overall improvement in preterm infant care.

Despite significant advances in perinatal medicine, caring for extremely preterm infants remains challenging due to their high mortality and morbidity risks. While survival rates have improved, these infants continue to face serious health issues. Effective management strategies, supported by meta-analyses and randomized controlled trials, are essential for reducing mortality and impairments. However, the efficacy and safety of some emerging strategies are still uncertain. Neonatal sepsis is a major concern, exacerbated by immature immune systems, prolonged hospitalizations, and frequent invasive procedures. Empiric broad-spectrum antibiotics are commonly used to treat sepsis, with therapy adjusted once pathogens are identified. Prophylactic maternal antibiotics and infection control measures, such as hand hygiene and central line care, are recommended. Nonetheless, overuse of antibiotics has led to increased rates of multidrug-resistant organisms and related complications, including bronchopulmonary dysplasia (BPD), necrotizing enterocolitis (NEC), and fungal infections. Furthermore, climate change, agricultural practices, occupational hazards, deforestation, human migration patterns, soil dispersion, patient immunosuppression, and advancements in infection detection and diagnostic testing all contribute to the rise of fungal diseases. The increasing rates of fungal morbidity and mortality are closely linked to antifungal resistance, drug tolerance, and biofilm formation [131]. Antifungal tolerance refers to the partial growth of fungi after 24 h of exposure to inhibitory drug concentrations, as evidenced in susceptibility tests. In contrast, antifungal resistance is characterized by the complete lack of a toxic effect on fungal pathogens despite treatment [132]. Current antifungal therapies are limited to a few drug classes, including polyenes, azoles, echinocandins, allylamines, and flucytosine. While allylamines are primarily used for superficial infections, the other four classes are highly effective against invasive mycosis. Moreover, evidence from randomized trials indicates that fluconazole prophylaxis may increase the risk of colonization with fluconazole-susceptible dose-dependent or resistant fungi. However, it does not significantly alter the risk of invasive infections caused by these fungi. The risk of breakthrough infections remains a concern and should be investigated further in large prospective studies [133]. Hence when managing a patient with or at risk for invasive fungal infections (IFI), clinicians must consider various factors to tailor therapy and optimize outcomes. Recent advancements have expanded antifungal options, each varying in spectrum, pharmacokinetics, indications, safety, cost, and usability. Matching these drug characteristics with patient-specific factors is crucial for effective treatment with minimal toxicity. Decision-making becomes especially complex for highly immunocompromised patients, such as ELBW neonates with rare IFIs, where a high level of suspicion is needed for early initiation of therapy. To achieve the best outcomes, extended antifungal treatment should be combined with addressing underlying risk factors and performing radical debridement of affected tissues.

## 5. Study Limitations

It is important to acknowledge the limitations of our study and interpret its findings with caution. The primary limitation is that our analysis relied exclusively on data from case reports and case series, which are known for their limited generalizability and validity, and their inability to establish causal relationships. Additionally, there may be significant biases related to publication, the retrospective nature of the study, and the focus on rare or atypical cases. However, some case reports and series can still provide valuable insights, especially in the absence of data from randomized controlled trials (RCTs) and observational studies. This is particularly relevant when a few studies suggest a significant and probable causal link in the context of an emerging epidemic or a newly introduced medication [134,135].

It should also be noted that due to the ongoing flux in the nomenclature of fungal agents, several *Candida* spp. have been renamed non-*Candida* genus, which are no longer considered causative agents for candidiasis. For example, *Candida pelliculosa* (now renamed to *Wickerhamomyces anomalus*/*Pichia anomala*) or *Candida norvegensis* (now renamed to *Pichia norvegensis*) [136].

In addition, significant neonatal risk factors were not available for all cases. There was also a lack of evidence on antifungal prophylaxis, antifungal susceptibility testing, and treatment administered. Moreover, fungemia-attributable mortality data was not available for all cases as well. Such gaps together with significant heterogeneity between review studies in recording baseline data were the main inhibiting factors for conducting a meta-analysis.

## 6. Conclusions

The present study has provided us with extended insight into the epidemiology of neonatal fungemia by non-*Candida* rare opportunistic yeasts among geographically different areas all over the world for an extended time period.

A rapid identification of these rare yeasts is a key element for adequate therapy and better outcomes for these vulnerable patients. Although recent advancements have paved the way with modern techniques and methodologies, fungemia by these opportunistic pathogens remains underestimated. Furthermore, it is obvious that the compliance of healthcare workers to general principles of hygiene is crucial in neonatal intensive care units. The problem is bigger in developing countries mainly due to a deficiency of resources.

## Figures and Tables

**Table 1:** Neonatal fungemia cases by rare opportunistic yeasts published in literature, since 1981.

Location	Year	Cases	Yeast Species	Identification Method	Reference
USA	1981	1 case	*Malassezia furfur*	NA	[8]
USA	1984	4 cases	*Malassezia furfur*	NA	[9]
USA	1985	3 cases	*Malassezia furfur*	NA	[10]
UK	1986	7 cases	*Hansenula anomala*	API 20C	[11]
USA	1987	5 cases	*Malassezia furfur*	NA	[12]
USA	1987	6 cases	*Malassezia furfur*	NA	[13]
USA	1989	3 cases	*Malassezia furfur*	NA	[14]
BELGIUM	1989	6 cases	*Malassezia furfur*	NA	[15]
NY	1991	1 case	*Malassezia furfur*	NA	[16]
USA	1991	2 cases	*Malassezia furfur*	NA	[17]
FRANCE	1991	1 case	*Malassezia furfur*	NA	[18]
USA	1992	1 case	*Trichosporon beigelii*	NA	[19]
EUROPE	1992	1 case	*Trichosporon asteroides*	NA	[20]
NY	1993	2 cases	*Trichosporon beigelii*	BacT/Alert	[21]
ATLANTA	1994	5 cases	*Malassezia pachydermatis*	NA	[22]
USA	1997	2 cases	*Trichosporon beigelii*	NA	[23]
USA	1997	1 case	*Cryptococcus laurentii*	NA	[24]
USA	1998	8 cases	*Malassezia pachydermatis*	Molecular	[25]
UK	1998	1 case	*Trichosporon beigelii*	API 32 ATB	[26]
CHANDIGARH	1999	10 cases	*Pichia anomala*	NA	[27]
SPAIN	2000	2 case	*Saccharomyces cerevisiae*	Vitek 2 API 20C Molecular	[28]
TAIWAN	2000	1 case	*Hansenula anomala*	NA	[29]
MALAYSIA	2000	1 case	*Hansenula anomala*	NA	[30]
INDIA	2001	33 cases	*Pichia anomala*	Morphology Biochemical tests	[31]
BRAZIL	2001	4 cases	*Pichia anomala*	NA	[32]
TAIWAN	2001	1 case	*Cryptococcus laurentii*	Morphology India ink Vitek 2	[33]
SWEDEN	2001	8 cases	*Malassezia pachydermatis*	NA	[34]
EUROPE	2002	1 case	*Trichosporon asahii*	API ID 32C	[35]
TURKEY	2003	1 case	*Trichosporon asahii*	NA	[36]
TURKEY	2003	3 cases	*Trichosporon mucoides*	API AUX C	[37]
ARGENTINA	2003	1 case	*Hansenula anomala*	API ID 32C	[38]
TURKEY	2004	1 case	*Pichia anomala*	API ID 32C	[39]
TURKEY	2005	1 case	*Saccharomyces cerevisiae*	NA	[40]
QATAR	2006	1 case	*Kodamaea ohmeri*	Vitek 2 API ID 32C Molecular	[41]
USA	2006	1 case	*Pichia fabianii*	Molecular	[42]
BRAZIL	2006	2 cases	*Pichia anomala*	Vitek 2	[43]
ITALY	2006	4 cases	*Rhodotorula mucilaginosa*	Vitek 2 API ID 32C	[44]
SOUTH KOREA	2007	1 case	*Kodamaea ohmeri*	Vitek 2 API20C	[45]
THAILAND	2008	1 case	*Cryptococcus neoformans*	NA	[46]
EUROPE	2008	1 case	*Trichosporon asahii*	Vitek 2	[47]
INDIA	2009	1 case	*Kodamaea ohmeri*	BacT/Alert 3D API ID 32C	[48]
BRAZIL	2009	1 case	*Trichosporon* spp.	Morphology	[49]
GREECE	2009	1 case	*Cryptococcus terreus*	Morphology MALDI-TOF MS Molecular	[50]
FRANCE	2010	1 case	*Pichia fabianii*	Molecular	[51]
INDIA	2011	1 case	*Rhodotorula mucilaginosa*	Morphology API 20C	[52]
ITALY	2011	1 case	*Malassezia furfur*	Morphology Molecular	[53]
KUWAIT	2011	1 case	*Kodamaea ohmeri*	Vitek 2 API 20C AUX Molecular	[54]
INDIA	2012	8 cases	*Trichosporon asahii*	Vitek 2	[55]
TAIWAN	2013	6 cases	*P. anomala (C. pelliculosa)*	API-32C Mini API Molecular	[56]
SOUTH AMERICA	2013	4 cases	*P. anomala (C. pelliculosa)*	Morphology Molecular	[57]
GREECE	2013	1 case	*Malassezia furfur*	Morphology MALDI-TOF MS Molecular	[50]
ITALY	2013	6 cases	*Malassezia furfur*	Vitek2 MALDI-TOF MS Molecular	[58]
TUNISIA	2013	1 case	*Saccharomyces cerevisiae*	NA	[59]
CHINA	2013	6 cases	*Pichia ohmeri*	Molecular	[60]
CHINA	2013	1 case	*Pichia fabianii*	API 20C AUX Molecular	[61]
CHINA	2013	1 case	*Kodamaea ohmeri*	API 20C AUX Vitek 2 Molecular	[62]
INDIA	2013	38 cases	*Kodamaea ohmeri*	Molecular	[63]
INDIA	2014	1 case	*Kodamaea ohmeri*	BacT/ALERT Vitek2	[64]
INDIA	2014	1 case	*Pseudozyma aphidis*	API ID 32C Vitek 2 Molecular	[65]
KUWAIT	2014	1 case	*Malassezia pachydermatis*	Vitek 2 MALDI–TOF MS Molecular	[66]
INDIA	2015	2 cases	*Kodamaea ohmeri*	Vitek 2 Molecular	[67]
CROATIA	2015	2 cases	*Cyberlindnera fabianii*	Molecular	[68]
ASIA	2015	3 cases	*Trichosporon asahii*	NA	[69]
NIGERIA	2015	1 case	*Moesziomyces bullatus*	Molecular	[70]
COLOMBIA	2016	1 case	*Kodamaea ohmeri*	Vitek 2	[71]
TURKEY	2017	1 case	*Wickerhamomyces anomalus*	Vitek 2	[72]
INDIA	2017	9 cases	*Pichia kudriavzevii*	Molecular	[73]
INDIA	2017	4 cases	*Rhodotorula glutinis*	NA	[74]
INDIA	2017	8 cases	*Cyberlindnera fabianii*	Molecular	[75]
INDIA	2017	1 case	*Wickerhamomyces anomalus*	Molecular	[75]
INDIA	2017	2 cases	*Saccharomyces cerevisiae*	Molecular	[76]
INDIA	2018	1 case	*Cryptococcus laurentii*	Vitek 2	[77]
PORTUGAL	2018	1 case	*Malassezia furfur*	MALDI–TOF MS	[78]
ITALY	2018	9 cases	*Malassezia furfur*	BacT/Alert	[79]
KUWAIT	2019	10 cases	*Cyberlindnera fabianii*	Molecular	[80]
TAIWAN	2019	1 case	*Malassezia furfur*	BacT/Alert	[81]
TAIWAN	2020	4 cases	*Malassezia pachydermatis*	BD BACTEC FX	[82]
CALIFORNIA	2020	3 cases	*Malassezia pachydermatis*	Molecular	[83]
INDIA	2020	12 cases	*Dirkmeia churashimaensis*	Molecular	[84]
INDIA	2020	2 cases	*Cyberlindnera fabianii*	MALDI-TOF-MS	[85]
KUWAIT	2021	1 case	*Papiliotrema laurentii*	Vitek 2 Molecular	[86]
COLOMBIA	2021	1 case	*Malassezia sympodialis*	Molecular	[87]
CHINA	2021	1 case	*P. anomala (C. pelliculosa)*	Molecular	[88]
CHINA	2021	21 cases	*P. anomala (C. pelliculosa)*	Vitek MS	[89]
CHINA	2021	14 cases	*P. anomala (C. pelliculosa)*	Molecular	[90]
GREECE	2022	1 case	*Moesziomyces aphidis*	Vitek 2 Molecular	[91]
KUWAIT	2022	1 case	*Lodderomyces elongisporus*	Vitek 2 Molecular	[92]
BRAZIL	2023	1 case	*Lodderomyces elongisporus*	Molecular	[93]
NIGERIA	2023	1 case	*Cryptococcus laurentii*	Vitek 2	[94]
INDIA	2023	1 case	*Aureobasidium melanogenum*	Molecular	[95]
INDIA	2023	1 case	*Kodamaea ohmeri*	Vitek 2 MALDI–TOF	[96]

NA Not available, API Analytical Profile Index, MALDI-TOF Matrix-assisted laser desorption/ionization time-of-flight, MALDI-TOF MS matrix-assisted laser desorption ionization-time of flight mass spectrometry.

**Table 2:** Cases per decade.

Years	Cases
1981–1990	35
1991–2000	39
2001–2010	69
2011–2023	199

**Table 3:** Risk factors (total cases/survival/exitus).

Risk Factors			
	Total Cases *n* = 342 NA Outcome *n* = 41	Survival *n* = 235/301 (78 %)	Exitus *n* = 66/301 (22%)
Low birth weight	335/342 (98%)	181/235 (77%)	55/66 (84%)
Central catheters	260/342 (76%)	178/235 (76%)	49/66 (74%)
Prematurity	226/342 (66%)	141/235 (60%)	44/66 (67%)
Broad spectrum antibiotics	222/342 (65%)	153/235 (65%)	46/66 (70%)
Parenteral nutrition	120/342 (35%)	89/235 (38%)	14/66 (21%)
Intubation/mechanical ventilation	116/342 (34%)	56/235 (24%)	43/66 (65%)
Sepsis	96/342 (28%)	63/235 (27%)	28/66 (43%)
Thrombocytopenia	72/342 (21%)	61/235 (26%)	14/66 (21%)
Prolonged hospitalization	61/342 (18%)	61/235 (26%)	28/66 (43%)
Respiratory distress syndrome	41/342 (12%)	28/235 (12%)	12/66 (19%)
Use of fluconazole	27/342 (8%)	9/235 (4%)	3/66 (5%)

NA (Not available) Outcome *n* = 41 (12%). Available Outcome *n* = 301 (88%).

**Table 4:** Antifungal susceptibility testing of species. Minimum inhibitory concentrations in (mg/L).

Yeast	Number of Isolates	AMB	FLC	VRC	POS	ITC	ISA	5-FC	AFG	MFG	CAS	Method Used	References
*Kodamaea ohmeri*	1	0.064	32	NA	NA	0.25	NA	<0.002	NA	NA	NA	Etest	[41]
	1	0.023	4	0.047	0.012	0.125		0.032	0.064	0.125	0.25	Etest	[54]
	38	0.25–1	0.5–64	0.03–8	0.06–4	0.06–4	NA	NA	NA	NA	0.12–1	CLSI	[63]
	2	0.25	4	0.25	NA	NA	NA	1	NA	NA	0.25	VITEK2	[67]
	1	<0.5	NA	NA	NA	NA	NA	<1	NA	NA	<0.25	VITEK2	[64]
*Cyberlindnera/Pichia fabianii*	1	0.03	8	NA	NA	NA	NA	0.125	NA	NA	NA	NCCLS	[42]
	1	0.5	2	NA	NA	NA	NA	0.25	NA	NA	NA	Etest	[51]
	8	0.75–12	0.5–16	NA	NA	NA	NA	NA	NA	NA	NA	Etest	[75]
	1	1	≤1	0.125	NA	2	NA	≤4	NA	NA	NA	CLSI	[61]
	2	NA	NA	NA	NA	NA	NA	NA	0.016–0.064	1–4	0.125–0.19	EUCAST	[68]
*Pichia anomala (Candida pelliculosa)*	2	0.023–0.032	3–4	0.064–0.125	NA	NA	NA	NA	NA	NA	NA	Etest	[43]
	33	NA	Only one isolate >64	NA	NA	NA	NA	NA	NA	NA	NA	CLSI	[31]
	6	1–2	0.125	NA	2	NA	NA	NA	NA	0.25–0.5	2	CLSI	[56]
	21	<0.5	2–4	0.125–0.25	NA	0.125–0.25	NA	<4	NA	NA	NA	CLSI	[89]
*Pichia kudriavzevii*	9	1	2–16	0.25	0.25–1	0.25–0.5	NA	NA	0.25–0.5	0.25–0.5	0.25–0.5	CLSI	[73]
*Moesziomyces (Pseudozyma)* *aphidis*	1	2	<0.125	8	0.03	0.03	≤0.016	>64	>8	>8	>8	EUCAST	[91]
*Moesziomyces (Pseudozyma) bullatus*	1	1	128	0.03	0.03	0.12	NA	64	8	8	8	Sensititre YeastOne Y010 microdilution method	[70]
*Dirkmeia (Pseudozyma) churashimaensis*	12	0.198	1 to 4	0.03–0.125	0.03–0.25	0.03–0.25	0.03–0.125	0.157	>8	>8	>8	CLSI	[84]
*Rhodotoroula mucilaginosa*	4	0.25	>256	2	NA	2	NA	0.125	NA	NA	NA	NA	[44]
	1	1.5	>256	0.38	NA	NA	NA	NA	NA	NA	>16	Etest	[52]
*Cryptococcus laurentii*	1	0.25	4	NA	NA	NA	NA	NA	NA	NA	NA	NCCLS	[33]
	1	0.25	2	NA	NA	NA	NA	NA	NA	NA	NA	NA	[77]
*Cryptococcus terreus*	1	0.125–1.5	128–256	0.125–0.5	0.25–0.5	NA	1	8–32	8–32	8–32	8–32	broth microdilution method Micronaut-AM and E-test	[50]
*Malassezia furfur*	1	0.19	0.25	0.094	0.094	0.125	0.5	16	32	32	32	MIC method on modified RPMI 1640 agar	[50]
*Malassezia pachydermatis*	1	0.19	>256	0.012	0.016	NA	NA	>32	NA	NA	>32	Etest	[66]

AMB: Amphotericin B, FLC: Fluconazole, VRC: Voriconazole, POS: Posaconazole, ITC: Itraconazole, ISA: Isavuconazole, 5-FC: Flucytosine, AFG: Anidulafungin, MFG: Micafungin, CAS: Caspofungin, NA: Not available, MIC: minimum inhibitory concentration, CLSI: Clinical and Laboratory Standards Institute, NCCLS: National Committee for Clinical Laboratory Standards, EUCAST: European Committee on Antimicrobial Susceptibility Testing.

**Table 5:** Treatment administered to neonates who survived and to whom died (survival/exitus). Amphotericin B (AmB), Fluconazole (FLC), Voriconazole (VOR), Miconazole (MIC), Caspofungin (CAS), 5-FC (5-flucytosine), KTC (ketoconazole).

Treatment	Survival	Exitus
CVC removal	21%	3%
Fluconazole (FLC)	30%	46%
Voriconazole (VOR)	5%	3%
Miconazole (MIC)	3%	2%
Caspofungin (CAS)	2%	0% or NA
Amphotericin B (AmB)	21%	33%
AmB + FLC	8%	10%
AmB + 5-FC (5-flucytosine)	4%	3%
AmB + FLC + 5-FC	4%	2%
AmB + KTC (ketoconazole)	1%	0% or NA

## Data Availability

Data are contained within the article.

## Supplementary Materials

The following supporting information can be downloaded at: https://www.mdpi.com/article/10.3390/ijms25179266/s1, Reference [137] is cited in the supplementary materials.
